# Supplementary material for: Assessing Related Factors of Intention to Perpetrate Dating Violence among University Students Using the Theory of Planned Behavior
Source: Int J Environ Res Public Health. 2020 Feb 2;17(3):923. doi: 10.3390/ijerph17030923 (PMC7036797; doi:10.3390/ijerph17030923)
Supplement: Supplementary file 1 [file ijerph-17-00923-s001.pdf]

Supplementary Material

Table 1. Items of the 26-item Dating Violence Behavioral Intention Questionnaire (DVBIQ).

TPB construct	Items /Scoring
**Direct measures**	**8 items**
Attitude	1. If I have a dating partner, my feeling to physically, mentally, or sexually abuse him/her in the following month is… *1 (Extremely bad) to 7 (Extremely good)*<br>2. In my opinion, physical, mental or sexual violence against a dating partner is… *1(Strongly unacceptable) to 7 (Strongly acceptable)*<br>3. In my opinion, intention to commit physical, mental or sexual violence against a dating partner is… *1(Strongly bad) to 7 (Strongly good)*
Subjective norm	1. Most of the people who influence me a lot (e.g., parents, friends, and teachers) hold an attitude that physical, mental, or sexual violence against a dating partner are…. *1 (Strongly disagree) to 7 (Strongly agree)*<br>2. Most of the people who influence me a lot (e.g., parents, friends, and teachers) think that physical, mental or sexual violence against a dating partner is… *1(Strongly unacceptable) to 7 (Strongly acceptable)*
Perceived behavior control	1. To me, physical, mental, or sexual violence against a dating partner in the following month is… *1 (Strongly not easy) to 7 (Strongly easy)*<br>2. The statement that in the following month I can commit physical, mental or sexual violence against a dating partner if I want is… *1 (Extremely incorrect) to 7 (Extremely correct)*
Intention	If I have a dating partner, the possibility of me perpetrating DV against him/her in the following month is… *1 (Strongly impossible) to 7 (Strongly possible)*
**Indirect measures**	**18 items** (9 composite items)
**Behavioral beliefs**	**Outcome evaluation**
1. Physical, mental, or sexual violence against dating partner will make him/her obey me… *1 (Strongly disagree) to 7 (Strongly agree)*<br>2. Physical, mental or sexual violence against the dating partner will release my emotion…	1. Physical, mental, or sexual violence against a dating partner can make him/her obey me, I feel this is... *1(Strongly bad) to 7 (Strongly good)*<br>2. Physical, mental or sexual violence against a dating partner can make me release my emotion, I feel this is… *1(Strongly bad) to 7 (Strongly good)*

Int. J. Environ. Res. Public Health **2020**, *17*, 923; doi:10.3390/ijerph17030923 www.mdpi.com/journal/ijerph

1 (Strongly disagree) to 7 (Strongly agree)	
**Normative beliefs**	**Motivation to comply**
1. Most of my family members (e.g., parents, brothers, sisters, etc.) think that physical, mental, or sexual violence against a dating partner is… *1 (Strongly unacceptable) to7 (Strongly acceptable)* 2. Most of my teachers think that physical, mental or sexual violence against a dating partner is… *1(Strongly unacceptable) to 7 (Strongly acceptable)* 3. Most of my classmates or friends think that physical, mental or sexual violence against a dating partner is… *1(Strongly unacceptable) to 7 (Strongly acceptable*	1. Will I follow my family's (parent, brother, sister) advice if I want to commit physical, mental, or sexual violence against dating partner…? *1 (Strongly unwilling to) to 7 (Strongly willing to)* 2. Will I follow my teachers' advice if I want to commit physical, mental or sexual violence against dating partner…? *1(Strongly unwilling to) to 7 (Strongly willing to)* 3. Will I follow my classmates' or friends' advice if I want to commit physical, mental or sexual violence against dating partner…? *1(Strongly unwilling to) to 7 (Strongly willing to)*
**Control beliefs**	**Power of control**
1. If the dating partner has a bad attitude (e.g., irresponsibility, disobedience, perfunctoriness, provocation, lying) that will provoke my physical, mental or sexual violence against a dating partner…. *1 (Strongly possible) to 7 (Strongly impossible)* 2. If the dating partner hangs out with the person I worry about without informing me, that will provoke my physical, mental or sexual violence against her/him. *1 (Strongly possible) to 7 (Strongly impossible)* 3. If I am unhappy or unable to control my emotion, that will provoke my physical, mental or sexual violence against a dating partner. *1 (Strongly possible) to 7 (Strongly impossible)* 4. My dating partner's disobedience will provoke my physical, mental or sexual violence against her/him. *1 (Strongly possible) to 7 (Strongly impossible)*	1. If the dating partner has a bad attitude (e.g., irresponsibility, disobedience, perfunctoriness, provocation, lying) that will provoke my physical, mental or sexual violence against her/him. I feel this to be… *1 (Strongly possible) to 7 (Strongly impossible)* 2. If the dating partner hangs out with the person I worry about without informing me that will provoke my physical, mental or sexual violence against her/him. I feel this to be… *1 (Strongly possible) to 7 (Strongly impossible)* 3. If I am unhappy or unable to control my emotion, that will provoke my physical, mental or sexual violence against a dating partner. I feel this to be… *1 (Strongly possible) to 7 (Strongly impossible)* 4. My dating partner's disobedience will provoke my physical, mental or sexual violence against her/him. I feel this to be… *1 (Strongly possible) to 7 (Strongly impossible)*

Note: TPB: Theory of Planned Behavior; PBC: Perceived behavioral control.
